# Characteristics of 2-drug regimen users living with HIV-1 in a real-world setting: A large-scale medical claim database analysis in Japan

**DOI:** 10.1371/journal.pone.0269779

**Published:** 2022-06-14

**Authors:** Daniel J. Ruzicka, Mayuko Kamakura, Naho Kuroishi, Nobuyuki Oshima, Miyuki Yamatani, Jingbo Yi, Bruce Crawford, Kunihisa Tsukada, Shinichi Oka

**Affiliations:** 1 Medical Affairs, MSD K.K., Tokyo, Japan; 2 Syneos Health, Tokyo, Japan; 3 AIDS Clinical Center, National Center for Global Health and Medicine, Tokyo, Japan; Harvard Medical School, UNITED STATES

## Abstract

**Background:**

Regimen simplification to 2-drug antiretroviral therapy (2-ART) may address potential tolerability issues, increase adherence, and reduce toxicity and potential drug-drug-interactions among people living with HIV-1 (PLWH). However, real-world treatment patterns and characteristics of 2-ART users are unclear.

**Methods:**

This retrospective observational cohort study employed a large-scale medical claim database of Japanese hospitals to extract data on 4,293 PLWH aged ≥18 years with diagnosis of HIV and treated with any ART regimens between April 2008 and April 2019. A 2-ART cohort was compared with a 3-drug antiretroviral therapy (3-ART) cohort in terms of population characteristics, comorbid conditions, and treatment patterns. Treatment switching rates were calculated for each cohort followed by sensitivity analysis to confirm the robustness of the findings.

**Results:**

There were 94 individuals identified in the 2-ART cohort. Compared to the standard 3-ART cohort (n = 3,993), the 2-ART cohort was older (median age 53 [IQR 44–64] vs 42 years [IQR 35–50]), with a lower proportion of males (87.2% vs 93.8%), higher Charlson Comorbidity Index (CCI) (median score 6 [IQR 5–8] vs 5 [IQR 4–6]), more co-medications (median 6 [IQR 4–11] vs 3 [IQR 2–7]), and a higher percentage of AIDS-defining conditions (66.0% vs 42.8%). The most common 2-ART were protease inhibitor (PI) + integrase strand transfer inhibitor (INSTI) and non-nucleoside reverse transcriptase inhibitor (NNRTI) + INSTI (33.0% and 31.9%, respectively). Overall, most of the regimens were nucleoside reverse transcriptase inhibitor (NRTI)-sparing (71.3%), with a decreasing trend over time (76.2% to 70.2%). ART regimen switch occurred more often in the 2-ART cohort than in the 3-ART cohort (33.0% vs 21.2%).

**Conclusion:**

The profiles of individuals on 2-ART in Japan were demonstrated to be complex. Most were treated with NRTI-sparing regimens which may reflect an effort to reduce treatment-related toxicities.

## Introduction

Antiretroviral therapy (ART) regimens have been credited with increasing the survival of people living with HIV-1 (PLWH) [1]. Three-drug ART regimens (3-ART), which consist of a backbone with two nucleoside reverse transcriptase inhibitors (NRTIs) plus an anchor drug with or without a booster, have become the standard of care (SoC) and treatment for HIV-1 [2, 3]. While advances in ART have been credited with improving survival by decades [4, 5], recent evidence of long-term adverse events (AEs) with 3-ART have been documented [6], particularly in terms of renal and bone toxicity [7–10] associated with treatment with tenofovir disoproxil fumarate (TDF). Although switches to tenofovir alafenamide (TAF) have been well-documented to result in relative less toxicity [11, 12], the extent of toxicity associated with TAF itself is inconclusive.

Further, rising concerns and studies of polypharmacy and potential drug-drug interactions have been well documented in complex regimens [13–16]. Protease inhibitors (PI) and certain integrase strand transfer inhibitors (INSTI) require additional administration with a booster such as ritonavir or cobicistat to increase their plasma concentrations. Ritonavir and cobicistat are strong non-specific inhibitors of cytochrome P450 (CYP) and other transporters involved in drug metabolism and pharmacokinetics [17, 18]. There is concern that ARTs containing a booster may cause further unknown drug interactions with other co-medications, resulting in toxicity or failure to reach therapeutic concentrations [19, 20]. As ART dosage and compatibility remain complex, such toxicities and complexities have prompted development of drug-sparing strategies with a simplified 2-drug ART regimen (2-ART) in attempt to reduce its negative impact.

In contrast, multi-center clinical trials of 2-ART (dolutegravir plus lamivudine and dolutegravir plus rilpivirine) have demonstrated to have non-inferior efficacy as well as safety compared to 3-ART [21, 22]. Based on the positive impact, there is an increasing diversity of therapies with a 2-drug combination of integrase strand transfer inhibitors (INSTI) plus protease inhibitors (PI) or non-nucleoside reverse transcriptase inhibitors (NNRTI). More ART regimens are being developed and used in real-world settings to tailor to the long-term complexities related to an individual’s genetic background, treatment history, and comorbidities [23].

In Japan, ART treatment uptake and clinical success among diagnosed HIV individuals, in terms of viral suppression, has been shown to be excellent and in line with UNAIDS/WHO targets of 90% [3, 24, 25]. However, Japanese PLWH have been demonstrated to have unique comorbidities and co-medication patterns [26]. There is limited evidence that switching to 2-ART may improve outcomes [27]. While the practice of regimen switching and reduction is an increasing global trend, the profile and number of individuals receiving 2-ART in real-world practice are not well understood in Japan. This study aimed to characterize individuals who received 2-ART and provide an overview of ART treatment patterns until April 2019 in Japan.

## Methods

### Study design and data source

This study was a retrospective cohort analysis of ART treatment patterns and population characteristics among adult individuals living with HIV on 2-ART compared to 3-ART in Japan. Data were extracted from a hospital-based medical claim database maintained by Medical Data Vision Co., Ltd. (MDV, Tokyo, Japan). The data is from all hospitals included in the MDV hospital panel, which covers 374 acute phase hospitals across regions (approximately 22% of acute phase hospitals in Japan) and approximately 25 million people. The MDV database includes treatment information, including antiretrovirals used, co-medications, and comorbidities, etc., but no laboratory results such as CD4 counts or plasma viral load.

### Study population

PLWH were included in the analyses if they had a diagnosis of HIV (ICD-10: B20-B24), had been treated with 2- or 3-ART, and were aged ≥18 years. The identification period for treatment index dates was from the first date available in the database (April 2008) to the last available (April 2019). An individual was considered to be on a 2-ART if they were prescribed any two antiretrovirals on the same day, and the first date of their 2-ART was the index date for this cohort. Individuals who did not have any 2-ART during their medical record were eligible for the 3-ART cohort if they were prescribed three antiretrovirals on the same day. The first date of their 3-ART was considered to be the index date for this cohort. Boosting agents (ritonavir or cobicistat) were not counted towards the 2-drug or 3-drug regimen definition and all individuals could only be classified into one cohort. Individuals were excluded if they were treated with a C-C chemokine receptor type 5 (CCR5) antagonist or two antiretrovirals in the same class as their index 2-ART because they are not SoC. Additionally, individuals treated with any regimens outside of 1) INSTI+2NRTI, 2) PI+2NRTI, or 3) NNRTI+2NRTI during their index 3-ART were excluded as they are also not SoC.

Demographic and clinical characteristics for the 2-ART cohort were stratified by three time periods to assess possible temporal changes due to novel drug access in Japan: April 1, 2008 to September 30, 2014 (representative of 1^st^ generation INSTI availability, mainly raltegravir), October 1, 2014 to June 30, 2016 (representative of 2^nd^ generation INSTI availability, including dolutegravir), and July 1, 2016 to April 30, 2019 (last available data period, representative of TAF availability as a novel prodrug of tenofovir to reduce toxicities). All drug classes and combinations were also tabulated for all individuals. To describe treatment characteristics, average gap (in days) between 2-ART and the total average duration of treatment were calculated from regimen initiation to last ART prescription in the database. Time to index regimen for the 2-ART cohort was calculated as the time from individuals’ first ART (prior to 2-drug initiation) to their index date.

### Outcomes

#### Population profiles

The analyses compared population characteristics on 2-ART with those with 3-ART. Population profiles consisted of demographics (age at index, gender, year of index date), co-medications, and comorbidities (hemophilia, AIDS-defining conditions, modified Charlson Comorbidity Index (CCI)). Modified CCI is severity indicator which is able to predict one-year mortality among hospitalized patients by weighting 17 different comorbid conditions (e.g. myocardial infarction, congestive heart failure), and has been validated using Japanese data [28]. Upon its validation, the CCI has been widely used as a comorbidity and severity indicator regardless of hospitalization status. All diseases and comorbidities were identified using ICD-10 codes or Japanese disease codes as available in the data. Additionally, all 2- and 3-drug combinations observed for each cohort were tabulated. These combinations were described by drug class: INSTI, PI, NNRTI, and NRTI. Regimens that did not contain any NRTIs were considered to be NRTI-sparing regimens in alignment with the definition of U.S. Department of Health and Human Services [29].

#### ART regimen switch

Two- and 3-ART switch patterns were also assessed from their index date. Individuals were considered to have switched their drug regimen if they had any change to their index regimen, such as an addition, removal, or exchange of a drug. Treatment switch rules were the same for both cohorts. However, it is noted that for individuals on 3-ART, they cannot switch to a 2-ART at any point by definition, since any evidence of 2-ART in an individual’s record would classify that person in the 2-ART cohort to begin with. In order to capture true switches, regimen changes were only counted as switches if they lasted for more than 90 days. Individuals on both 2- and 3-ART were considered to have switched even if they remained on the same number of drugs, as long as a change in any component of their regimen was present. Specific transitions were not considered switches: the same regimen with only tenofovir disoproxil fumarate (TDF) switched to TAF, the same regimen with changes in dosing, or the same regimen with an added/switched booster. Switch rate was defined as number of individuals who switched divided by total person years. Total person-years for treatment switches was time from index treatment initiation to the first treatment switch, whereas person-years for individuals who did not switch was defined as time from the first treatment initiation to the last ART prescription during the study period. The switch rate and time-to-next-switch were calculated from the index date to the individual’s next treatment switch, the last ART claim in the database, or end of the study period, whichever occurs first. The switch rate observed in among both cohorts were compared.

Finally, a sensitivity analysis of switching was performed to make comparisons among individuals with at least one switch and with similar follow-up time after the switch. As described in **S1 Fig**, only individuals on 2-ART with at least one antiretroviral drug prior to their index date and individuals on 3-ART with at least 1 switch after their index date were included to ensure that the starting point of switch analysis would be after at least one switch and only assessed for treatment experienced individuals. In addition, only individuals with at least one year of follow-up data were included to ensure that potential switches could be captured in the data. In the switch sensitivity analyses, the switch index date was set to patient’s first switch for the 3-ART cohort and the switch rate was calculated from the time of the first switch to their next switch.

### Statistical analysis

All statistical analysis was completed on SAS version 9.4 (SAS Institute Inc., Cary, USA). All descriptive data were presented as frequencies for categorical variables and mean (standard deviation [SD]) or median [Q1, Q3] for continuous variables. Comparisons were made using the chi-square and t-tests, respectively. Time-to-first switch is presented using Kaplan-Meier curves and 95% confidence limits of the switch rate were calculated assuming a Poisson distribution. Multivariable analyses were conducted to explore the differences in comedications, AIDS-defining conditions, and CCI score using age, gender, and index year cluster as covariates. Logistic regression was utilized in a model for any comedications and AIDS-defining conditions, and linear regression was utilized for the CCI score.

### Ethics approval and consent to participate

The study protocol was reviewed and approved by the Rinsyou Kenkyuu Suishin Network Japan Ethics Committee (Approval Number: 518-NIS-8422). The Japanese Ethical Guidelines for Medical and Health Research Involving Human Subjects (http://www.mhlw.go.jp/file/06-Seisakujouhou-10600000-Daijinkanboukouseikagakuka/0000153339.pdf) do not apply to studies exclusively using de-identified data, so we were not obligated to obtain informed consent in this study.

## Results

### Study sample

**Fig 1:**
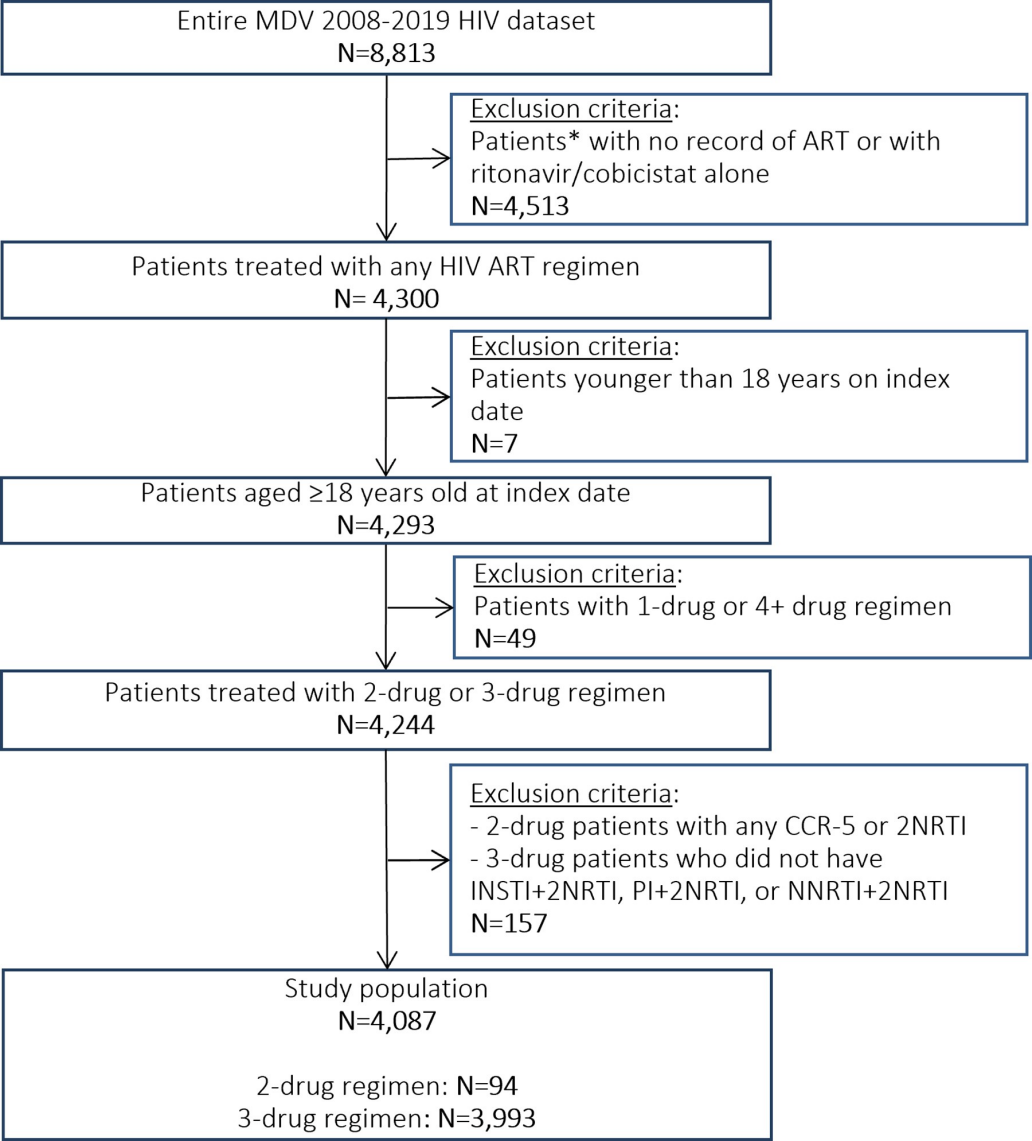
Flow chart of cohort attrition. *Individuals in this study were selected from a hospitals-based database, thus are referred to as “patients” in this context.

For the study period between April 1, 2008, and April 30, 2019, a total of 8,813 individuals with a diagnosis of HIV were identified. Among them, 4,513 cases were excluded because of lack of an ART record. From the remaining 4,300 cases, all eligible cases were selected according to inclusion/exclusion criteria and a final 4,087 individuals were analyzed in this study. The flow of the process was illustrated in **Fig 1.** Among the included individuals, 94 (2.3%) received a 2-ART on their index date and were classified as the 2-ART cohort. There were 3,993 (97.7%) individuals who received a 3-ART and were classified as the 3-ART cohort. The median duration of follow-up was 712 days and 701 days in the 2-ART cohort and the 3-ART cohort, respectively.

### Baseline and clinical overview

Of the 94 individuals in the 2-ART cohort, the median age (IQR) was 53 years (44, 64 years) and 87.2% were male. In contrast, the 3-ART cohort was significantly younger with the median age (IQR) of 42 years (35, 50 years) and included significantly more males (93.8%). The proportion of individuals having hemophilia was greater in the 2-ART cohort than in the 3-ART cohort (12.8% vs. 2.1%; p<0.001) (**Table 1**).

**Table 1:** Baseline characteristics.

	2-Drug		3-Drug		P-value
**Demographics (at Index)**					
Age	94		3993		<0.01
Median (Q1, Q3)	53 (44, 64)		42 (35, 50)		
Min, max	27,82		18,90		
Gender (n, %)					
Male	82	87.2%	3745	93.8%	0.01
Female	12	12.8%	248	6.2%	
Hemophilia (n, %)					
Yes	12	12.8%	84	2.1%	<0.01
Year of index date (n, %)					
Cluster 1 (April 1, 2008- September 30, 2014)	21	22.3%	1057	26.5%	<0.01
Cluster 2 (October 1, 2014- June 30, 2016)	26	27.7%	420	10.5%	
Cluster 3 (July 1, 2016—April 1, 2019)	47	50.0%	2516	63.0%	
**Clinical background Summary**					
Co-medications					
N with at least other 1 medication class	87	92.6%	2996	75.0%	<0.01
Median (Q1, Q3)	6 (4, 11)		3 (2, 7)		<0.01
Number of AIDS-defining conditions					
N with at least 1 condition	62	66.0%	1708	42.8%	<0.01
Median (Q1, Q3)	2 (1, 2)		1 (1, 2)		0.69
0	32	34.0%	2285	57.2%	
1	30	31.9%	919	22.0%	
2	21	22.3%	443	11.1%	
3+	11	11.7%	346	8.7%	
Charlson Comorbidity Index	94		3993		
Median (Q1, Q3)	6 (5,8)		5 (4,6)		<0.001
Charlson comorbid conditions					
Congestive Heart failure	25	26.6%	202	5.1%	<0.01
Dementia	0	0.0%	17	0.4%	N/A
Chronic pulmonary disease	28	29.8%	940	23.5%	0.16
Rheumatologic disease	4	4.3%	49	1.2%	0.03
Mild liver disease	41	43.6%	1330	33.3%	0.04
Diabetes with chronic complications	18	19.2%	163	4.1%	<0.01
Hemiplegia or paraplegia	2	2.1%	30	0.8%	0.17
Renal disease	32	34.0%	192	4.8%	<0.01
Any malignancy, including lymphoma and leukemia	16	17.0%	294	7.4%	<0.01
Moderate or severe liver disease	5	5.3%	23	0.6%	<0.01
Metastatic solid tumor	2	2.1%	37	0.9%	0.23
HIV	94	100.0%	3990	99.9%*	N/A
Other relevant systemic diseases					
Hemodialysis (artificial kidney, chronic maintenance dialysis)	6	6.4%	6	0.2%	<0.01
Cardiovascular diseases	12	12.8%	178	4.5%	<0.01
Hypertension	50	53.2%	765	19.2%	<0.01
Lipid disorders	40	42.6%	711	17.8%	<0.01
Bone Disorders	17	18.1%	251	6.3%	<0.01
Peripheral Neuropathy	0	0.0%	0	0.0%	N/A
Any Diabetes	48	51.1%	871	21.8%	<0.001
Psychiatric disorders	39	41.5%	1446	36.2%	0.29
Hepatitis B/C Co-Infection	24	25.5%	878	22.0%	0.41

*Although all individuals included in the analysis had an HIV diagnosis code, not all 3-drug individuals had a diagnosis for HIV using the Charlson Comorbidity HIV codes, which do not include B23 (*HIV disease resulting in other conditions*), yet was used for the study population definition.

### Co-medications

Of individuals in the 2-ART cohort, 92.6% had been prescribed at least one class of co-medication with the median (IQR) of 6 different co-medication classes (4, 11 classes) (based on ATC level 4 code) during the follow-up period. The top five most common co-medication classes were proton pump inhibitors, angiotensin II antagonists, calcium antagonists, anti-gout preparations, and non-barbiturates and statins [**S1 Table**]. In contrast, 75.0% of individuals in the 3-ART cohort have been prescribed a co-medication, with a lower median (IQR) number of different co-medication classes of 3 (2, 7 classes) (p<0.01) [Table 1, **S2 Fig**]. The most commonly prescribed co-medications in the 3-ART cohort included systemic antihistamines, non-barbiturates, proton pump inhibitors, non-steroidal anti-rheumatics, and other anti-ulcerants. After accounting for potential differences in age, gender, and year of initiation, the 2-ART cohort remained more likely to have at least 1 comedication compared to the 3-ART cohort [**S2 Table**].

### AIDS-defining conditions

AIDS-defining conditions varied between the 2- and 3-ART cohorts; 66.0% of individuals on 2-ART had an AIDS-defining condition compared with 42.8% of those on 3-ART [**S3 Table**]. AIDS-defining conditions other than an AIDS diagnosis were present in 42.6% and 31.6% of individuals in the 2- and 3-ART cohorts, respectively. A diagnostic code for AIDS itself was found in 52.1% and 24.8% of the 2- and 3-ART cohorts, respectively. The top 5 most common AIDS-defining conditions other than AIDS itself was the same for both cohorts: pneumocystis (20.2% and 22.1%), cytomegalovirus disease (17.0% and 10.5%), tuberculosis (6.4% and 6.4%), non-Hodgkin lymphoma (4.3% and 3.2%), and encephalopathy (4.3% and 2.5%). After accounting for potential differences in age, gender, and year of initiation, the 2-ART cohort remained significantly more likely to have at least 1 AIDS-defining compared to the 3-ART cohort [**S3 Table**]. Similar to comedications, older age and earlier year cluster of initiation were also observed to be significantly associated with having an AIDS-defining condition.

### Comorbidities

Nearly all Charlson comorbid conditions were more prevalent in the 2-ART cohort than in the 3-ART cohort (median score 6 vs. 5), with the largest differences found for mild liver disease (43.6% vs. 33.3%), congestive heart failure (26.6% vs. 5.1%), renal disease (34.0% vs. 4.8%), diabetes with chronic complications (19.2% vs. 4.1%), and malignancies (17.0% vs. 7.4%) (**Table 1**). In addition, the 2-ART cohort also showed a significantly higher prevalence of all other relevant systemic diseases, except for psychiatric disorders and hepatitis B/C co-infection. In multivariable analysis, the difference in CCI score remained significantly higher for the 2-ART cohort, older age, males, and 1^st^ index year cluster (compared to last year cluster).

### Treatment patterns

**Fig 2:**
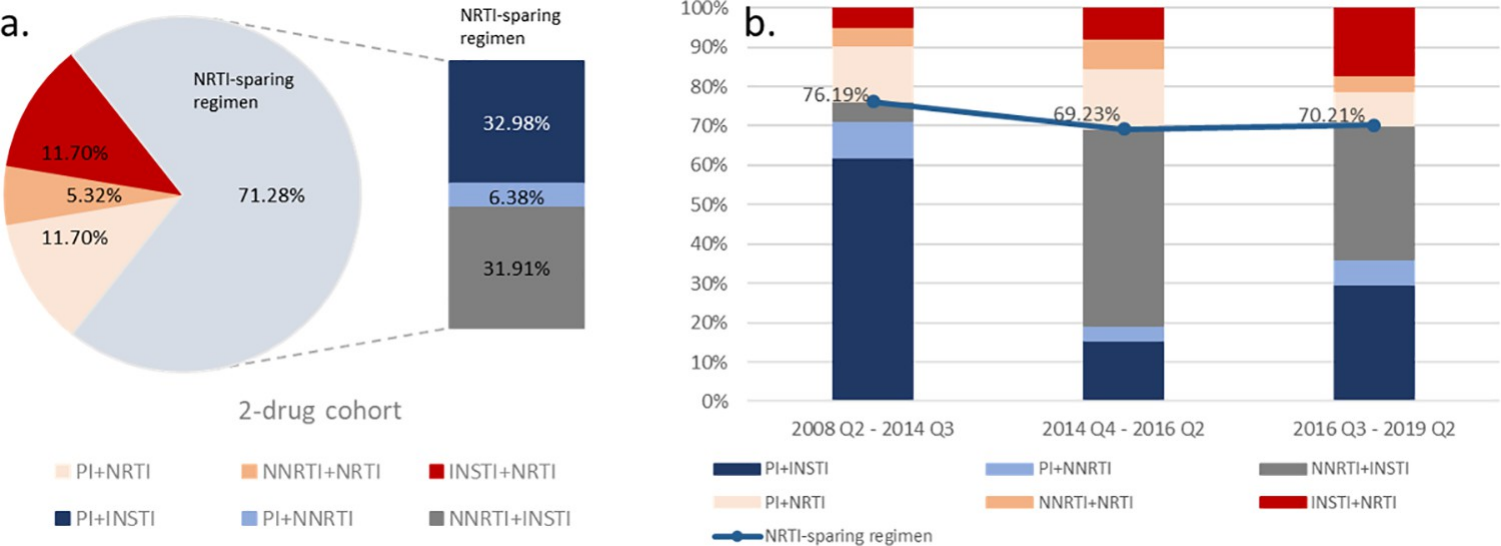
Analysis of 2-ART drug regimens. (a.) Treatment patterns by drug combination regimen at index date. (b.) Drug combinations over three periods for the 2-drug cohort.

Among individuals on a 2-ART, NRTI-sparing regimens comprised the majority of all 2-ARTs (71.3%) (**Fig 2A**). Overall, the most common class combinations for this cohort were PI+INSTI (33.0%) and NNRTI+INSTI (31.9%). Among those treated with a 3-ART, the most common drug class combinations were INSTI+2NRTI (66.6%), followed by PI+2NRTI (20.5%) and NNRTI+2NRTI (12.9%). The top common antiretroviral drug combinations for both cohorts are presented in [**S4 Table**].

The 2-ART cohort comprised of 1.9% of all individuals from April 1, 2008 to September 30, 2014 (representative of 1^st^ generation INSTI), 5.8% between October 1, 2014 and June 30, 2016 (representative of 2^nd^ generation INSTI), and 1.8% between July 1, 2016 and April 30, 2019 (representative of TAF). Among those in the 2-ART cohort, NRTI-sparing regimens were frequently found in all time periods examined (**Fig 2B**). Within the 2-ART cohort, the first time period (April 1, 2008 to September 30, 2014) had the highest proportion of NRTI-sparing regimens (76.2%), with the proportion decreasing slightly to 69.2% in the second time period (October 1, 2014 to June 30, 2016), then leveling out at 70.2% in the last time period (July 1, 2016 to April 30, 2019). Over time, there was a shift in drug class among NRTI-sparing regimens among the 2-ART cohort. Specifically, PI+INSTI (61.9%) regimens were dominant in the first time period (April 1, 2008 to September 30, 2014), but NNRTI+ INSTI regimens were most common from October 1, 2014 to April 30, 2019.

### Switch analyses

ART regimen switch occurred more often in the 2-ART cohort (33.0%; 31/94) than in the 3-ART cohort (21.2%; 845/3993). The switch rate was 20.88 switches/100 person-years (95% CI: 14.68–29.68) in the 2-ART cohort, and 10.34 switches/100 person-years (95% CI: 9.66–11.06) in the 3-ART cohort; however, no statistical comparisons were made (**Table 2**). In the time-to-switch analysis, around 25% of individuals in the 2-ART cohort had their first switch within one year, while only 10% of individuals in the 3-ART cohort had their first switch within one year. As presented in the Kaplan-Meier curve, the higher switch rate in the 2-ART cohort was most evident prior to year 5 of follow-up. However, due to the small cohort sample, long-term switch rates were more difficult to evaluate [**S3 Fig**].

**Table 2:** Treatment switching patterns.

	N	Switch rate (/100 person years)	Lower CI	Upper CI
Any Switch	876	10.5	9.9	11.2
2-Drug regimen cohort	31	20.9	14.7	29.7
2-Drug to 2-drug switches	3			
2-Drug to 3-drug switches	24			
2-Drug to 4-drug switches	4			
3-Drug regimen cohort	845	10.3	9.7	11.1
3-Drug to 3-drug switches	832			
3-Drug to 4-drug switches	9			
3-Drug to 1-drug switches	4			
*Switch sensitivity analyses* *				
Any Switch	130	6.4	5.4	7.6
2-Drug regimen cohort	19	29.2	18.7	45.8
2-Drug to 2-drug switches	2			
2-Drug to 3-drug switches	13			
2-Drug to 4-drug switches	4			
3-Drug regimen cohort**	111	5.7	4.7	6.8
3-Drug to 3-drug switches	107			
3-Drug to 4-drug switches	2			
3-Drug to 1-drug switches	2			
Total person-years (PY) for any switch		**PY**		
2-Drug	94	149		
3-Drug	3,393	8,176		
Follow-up time, days		**Median**	**Q1**	**Q3**
2-Drug	94	712	351	1,264
3-Drug	3,393	701	552	1,361

*Subset of individuals with at least 1 year follow-up and 1 prior switch

**Only individuals who additionally switched to another 3-drug regimen were included.

Of all switches in the 2-ART cohort, 77.4% (24/31) was from 2-ART to 3-ART (**Table 2**). All switch patterns for the 2-ART cohort are presented in [**S4 Fig**]. The most common switching patterns were from PI+NRTI to PI+2NRTI and PI+INSTI to INSTI+2NRTI. Only 9.7% (3/31) of all switches in the 2-ART cohort were from a 2-ART to another 2-ART. In the 3-ART cohort, because individuals could not switch to a 2-ART by definition, 98.5% (832/845) of all switches were to another 3-ART (rather than to a 1- or 4-drug regimen). The most prevalent switch pattern was from PI+2NRTI to INSTI+2NRTI. About 1% of 3-ART cohort who switched changed to a 4-drug regimen, such as adding a PI to INSTI+2NRTI regimen.

Sensitivity analysis demonstrated that in the subset of individuals with at least 1 year follow-up and 1 prior switch at the starting point of assessment, the switch rate (per 100 person-years (PY)) was higher among the 2-ART cohort (29.24/PY; 95% CI: 18.65–45.84/PY) compared to the 3-ART cohort (5.67/PY; 95% CI: 4.71–6.83/PY).

## Discussion

Although real-world studies of 2-drug regimens exist [30–33], this is the first study in Japan to assess characteristics of PLWH on 2-drug regimens compared with those on the standard 3-ART regimens in a real-world setting to our knowledge. Despite the small number of individuals in the 2-ART cohort, and that they were more experienced at the index date, individuals on a 2-ART were significantly older, received a larger number of co-medications, and had a higher CCI score as well as higher proportion of individuals with AIDS-defining conditions, compared with individuals on a 3-ART.

These characteristics suggest that disease status was more severe and complicated in individuals on a 2-ART. In addition, hypertension, diabetes, lipid disorders, renal disease and congestive heart failure were significantly higher in the 2-ART cohort compared with 3-ART cohort. Similar comorbidities were highlighted in a review of multi-comorbidities in PLWH [34]. It was also proposed in the review that clinical reassessment be scheduled for at-risk individuals, defined as those who are older than 50 years, have 2 or more comorbidities or receiving polypharmacy (generally considered as 6 or more medications). Another study also indicated that multi-comorbidities were higher in the 2-ART cohort compared with 3-ART cohort [30]. They also found that individuals initiating 2-ART were older and had shorter time-to-discontinuation [30]. These differences, especially older age and more comorbidities, are also well established in recent real world comparisons [31–33]. The significantly higher proportion of renal disease, hemodialysis and bone disorders in the 2-ART cohort compared with 3-ART cohort might be a reason for the choice of NRTI-sparing regimen. Some studies have demonstrated that greater decrease in renal function and bone mineral density (BMD) were observed in subjects treated with TDF-containing regimens than were observed in subjects treated with TDF-sparing regimens. TDF was well known to cause proximal tubular and renal toxicities, especially in individuals with low body weight not only in Japanese but also in Vietnamese populations [35–37], and were irreversible after a certain period of TDF use [38]. NRTI-sparing regimen comprised the majority of all 2-ARTs in our study, which might suggest that TDF-sparing strategies were adopted to avoid these toxicities. Thus, resorting to non-standard regimens based on individuals’ more complex and sicker profiles reflect the strategy that we refer to as “negative selection”. In contrast, individuals who are not as sick as the population identified in our study may be also placed on 2-ART regimens an option, especially for naïve individuals or treatment-experienced individuals with few complications as these could be switches for convenience. We refer to 2-ART regimen selection of this nature as “positive selection”.

When stratifying by time periods that reflected the commercial availability of specific antiretrovirals in Japan, there was a shift in drug class among the NRTI-sparing regimens from PI + INSTI regimens to NNRTI + INSTI regimens between 2008 to 2019. With increase in variety of ART treatment options, 2-ART combinations seemed to be personalized to individuals and have become diverse. Accordingly, the shift to NNRTI + INSTI as represented by “dolutegravir + rilpivirine” has increased as a new NRTI-sparing regimen. The most common switching pattern of NRTI-sparing regimen with PI + INSTI to standard 3-ART with INSTI + 2NRTI might have occurred because a randomized study (NEAT001/ANRS143) suggested that “raltegravir + darunavir/ritonavir” group have more failures in individuals with low baseline CD4 cell count [39]. Since this is a retrospective study and contains only data up to April 2019, as the landscape of 2-drug therapy is shifting in Japan, the treatment patterns and utilization profiles of 2-ART also need to be revisited as more data become available, especially with the approval of dolutegravir sodium/rilpivirine hydrochloride in late 2018 and the approval of dolutegravir sodium/lamivudine in 2020.

In the current study, individuals in the 2-ART had a switch rate approximately two times higher than the 3-ART cohort, indicating some potential challenges in managing individuals on 2-ART. Our study has clearly characterized individuals on 2-ART might be difficult to treat regardless of 2 or 3-ART. Several studies on 2-ART have demonstrated heterogeneous efficacy results [40–42], some demonstrating inferiority compared to SoC. However, recently several 2-ARTs have demonstrated non-inferiority and have been approved in several countries. Since these studies were usually randomized controlled trials with a very controlled patient population, data from real-world settings will need to demonstrate the robustness of these regimens. Although the MDV database is not completely representative of the general Japanese HIV population due to its use of acute hospital data, this study objective was to compare 2-ART and 3-ART regimens, where both groups were identified in the same database. We assume that a similar relationship between the two comparison groups would hold in the general population as well.

There are several limitations inherent in database studies. The MDV database is not representative of all PLWH in general as described. Doctors’ choices of ART regimens or switching strategies may also differ not only among hospitals in the database but also between these hospitals and HIV-specialized facilities. It is also not possible to track individuals as they move between hospitals. Individuals may only receive an HIV diagnosis in an MDV hospital during a medical encounter, but received treatment elsewhere, as observed in cohort attrition from the large drop between HIV diagnoses captured in the database and individuals receiving ART. Similarly, individuals may be considered treatment-naïve based on their first observed therapy in MDV data, but may have received treatment elsewhere. To address this shortfall, sensitivity analysis was performed that required individuals to meet criteria for inclusion as “continuously” receiving care and assessed individuals from their first captured switch in the database. There may still be differential follow-up time between 2-ART and 3-ART cohort beyond one year, a potential remaining source of bias when evaluating switch rates. Limitations also exist with the switch analysis for the 3-ART cohort as, due to limited sample size, any individual on a 2-antiretroviral was classified into the 2-ART cohort. Therefore, a 3-ART switch could not drop to a 2-ART. The causes for switching could not be captured in this retrospective database study, which limits the interpretability and extent of clinical implications of our findings. In addition, with the 2-ART cohort, the index date is the date of initiation of 2-ART, which could be quite far along in their treatment course. Similarly, confounding by indication in assignment to 2-ART and 3-ART regimens cannot be excluded, as concerns regarding drug-drug interactions or the severe disease status of 2-ART are more likely drivers for their regimen assignment. Finally, lab results, such as viral load and CD4+ counts, were not recorded in the database, only the number of the test was recorded. Therefore, the clinical effects of 2- versus 3-ART could not be assessed.

This study was the first step in understanding population profiles and medical needs of those who benefit from 2-ART in Japan. It provides a foundation for future studies to further analyze and explore reasons for switching, as well as identifying those may most benefit from NRTI-sparing regimen. As it is expected that more individuals will receive 2-ART by positive selection in the future, research characterizing the benefits of these regimens is imperative.

## Conclusion

In conclusion, PLWH in Japan on 2-ART demonstrated to have complex profiles, were older, and had more comorbidities and co-medications compared to individuals on 3-ART. This highlighted the negative selection of 2-ART regimens and the need for careful management by physicians to reduce treatment-related toxicities until April 2019.

## Supporting information

S1 Table Rank of co-medications. (DOCX)

S2 Table Adjusted analysis of co-medications and comorbidities. Age was included as a continuous variable and the corresponding OR represents per 1-year increase.
(DOCX)

S3 Table AIDS-defining conditions among each cohort. All conditions were identified using ICD-10 codes or Japanese disease codes.
(DOCX)

S4 Table Top 10 regimen in 2- and 3-drug regimen cohort. All regimens listed above may or may not be used with an additional booster; *Tenofovir includes tenofovir disoproxil fumarate and tenofovir alafenamide fumarate.
(DOCX)

S1 Fig Study design. (DOCX)

S2 Fig Number of co-medications. (DOCX)

S3 Fig Kaplan-Meier curve for time to switch for the 2- and 3-drug regimen cohorts. (DOCX)

S4 Fig Switch patterns for 2-drug regimen cohort. (DOCX)

## References

[1] Antiretroviral Therapy Cohort Collaboration. Survival of HIV-positive patients starting antiretroviral therapy between 1996 and 2013: a collaborative analysis of cohort studies. Lancet HIV. 2017;4(8): e349–e56. doi: 10.1016/S2352-3018(17)30066-8 28501495 PMC5555438

[2] Nation Institutes of Health. Guidelines for the Use of Antiretroviral Agents in Adults and Adolescents with HIV. 2019. [cited 2020 March 4]. Available from: https://files.aidsinfo.nih.gov/contentfiles/lvguidelines/AA_Recommendations.pdf.

[3] Consolidated guidelines on the use of antiretroviral drugs for treating and preventing HIV infection: recommendations for a public health approach. 2016. [cited 2020 March 4]. Available from: https://apps.who.int/iris/bitstream/handle/10665/208825/9789241549684_eng.pdf?sequence=1&isAllowed=y. 27466667

[4] Teeraananchai S, Kerr SJ, Amin J, Ruxrungtham K, Law MG. Life expectancy of HIV-positive people after starting combination antiretroviral therapy: a meta-analysis. HIV Med. 2017;18(4): 256–66. doi: 10.1111/hiv.12421 27578404

[5] Oka S, Ikeda K, Takano M, Ogane M, Tanuma J, Tsukada K, et al. Pathogenesis, clinical course, and recent issues in HIV-1-infected Japanese hemophiliacs: a three-decade follow-up. Glob Health Med. 2020;2(1): 9–17. doi: 10.35772/ghm.2019.01030 33330768 PMC7731362

[6] Chawla A, Wang C, Patton C, Murray M, Punekar Y, de Ruiter A, et al. A Review of Long-Term Toxicity of Antiretroviral Treatment Regimens and Implications for an Aging Population. Infect Dis Ther. 2018;7(2): 183–95. doi: 10.1007/s40121-018-0201-6 29761330 PMC5986685

[7] Horberg M, Tang B, Towner W, Silverberg M, Bersoff-Matcha S, Hurley L, et al. Impact of Tenofovir on Renal Function in HIV-Infected, Antiretroviral-Naive Patients. J Acquir Immune Defic Syndr. 2010;53(1): 62–9. doi: 10.1097/QAI.0b013e3181be6be2 19838127

[8] Kinai E, Hanabusa H. Progressive renal tubular dysfunction associated with long-term use of tenofovir DF. AIDS Res Hum Retroviruses. 2009;25(4): 387–94. doi: 10.1089/aid.2008.0202 19361280

[9] McComsey GA, Kitch D, Daar ES, Tierney C, Jahed NC, Tebas P, et al. Bone mineral density and fractures in antiretroviral-naive persons randomized to receive abacavir-lamivudine or tenofovir disoproxil fumarate-emtricitabine along with efavirenz or atazanavir-ritonavir: Aids Clinical Trials Group A5224s, a substudy of ACTG A5202. J Infect Dis. 2011;203(12): 1791–801. doi: 10.1093/infdis/jir188 21606537 PMC3100514

[10] Stellbrink HJ, Orkin C, Arribas JR, Compston J, Gerstoft J, Van Wijngaerden E, et al. Comparison of changes in bone density and turnover with abacavir-lamivudine versus tenofovir-emtricitabine in HIV-infected adults: 48-week results from the ASSERT study. Clin Infect Dis. 2010;51(8): 963–72. doi: 10.1086/656417 20828304

[11] Tao X, Lu Y, Zhou Y, Zhang L, Chen Y. Efficacy and safety of the regimens containing tenofovir alafenamide versus tenofovir disoproxil fumarate in fixed-dose single-tablet regimens for initial treatment of HIV-1 infection: A meta-analysis of randomized controlled trials. Int J Infect Dis. 2020;93: 108–17. doi: 10.1016/j.ijid.2020.01.035 31988012

[12] Squillace N, Ricci E, Menzaghi B, De Socio GV, Passerini S, Martinelli C, et al. The Effect of Switching from Tenofovir Disoproxil Fumarate (TDF) to Tenofovir Alafenamide (TAF) on Liver Enzymes, Glucose, and Lipid Profile. Drug Des Devel Ther. 2020;14: 5515–20. doi: 10.2147/DDDT.S274307 33364747 PMC7751319

[13] Atta MG, De Seigneux S, Lucas GM. Clinical Pharmacology in HIV Therapy. Clin J Am Soc Nephrol. 2019;14(3): 435–44. doi: 10.2215/CJN.02240218 29844056 PMC6419290

[14] Vivithanaporn P, Kongratanapasert T, Suriyapakorn B, Songkunlertchai P, Mongkonariyawong P, Limpikirati PK, et al. Potential drug-drug interactions of antiretrovirals and antimicrobials detected by three databases. Scientific Reports. 2021;11(1): 6089. doi: 10.1038/s41598-021-85586-8 33731842 PMC7971054

[15] Devanathan AS, Anderson DJC, Cottrell ML, Burgunder EM, Saunders AC, Kashuba ADM. Contemporary Drug-Drug Interactions in HIV Treatment. Clin Pharmacol Ther. 2019;105(6): 1362–77. doi: 10.1002/cpt.1393 30739315

[16] Bastida C, Grau A, Márquez M, Tuset M, De Lazzari E, Martínez E, et al. Polypharmacy and potential drug-drug interactions in an HIV-infected elderly population. Farm Hosp. 2017;41(5): 618–24. doi: 10.7399/fh.10778 28847251

[17] Tseng A, Hughes CA, Wu J, Seet J, Phillips EJ. Cobicistat Versus Ritonavir: Similar Pharmacokinetic Enhancers But Some Important Differences. Ann Pharmacother. 2017;51(11): 1008–22. doi: 10.1177/1060028017717018 28627229 PMC5702580

[18] Marzolini C, Gibbons S, Khoo S, Back D. Cobicistat versus ritonavir boosting and differences in the drug-drug interaction profiles with co-medications. J Antimicrob Chemother. 2016;71(7): 1755–8. doi: 10.1093/jac/dkw032 26945713

[19] Hollywood P, MacCann R, Lorigan D, de Barra E, McConkey S. Pharmacokinetic enhancers (cobicistat/ritonavir) and the potential for drug-drug interactions. Ir J Med Sci. 2020;189(2): 693–9. doi: 10.1007/s11845-019-02125-1 31735989

[20] Vélez-Díaz-Pallarés M, Esteban-Cartelle B, Montero-Llorente B, Gramage-Caro T, Rodríguez-Sagrado M, Bermejo-Vicedo T. Interactions of cobicistat and ritonavir in patients with HIV and its clinical consequences. Enferm Infecc Microbiol Clin (Engl Ed). 2020;38(5): 212–8. doi: 10.1016/j.eimc.2019.09.009 31753469

[21] Cahn P, Madero JS, Arribas JR, Antinori A, Ortiz R, Clarke AE, et al. Dolutegravir plus lamivudine versus dolutegravir plus tenofovir disoproxil fumarate and emtricitabine in antiretroviral-naive adults with HIV-1 infection (GEMINI-1 and GEMINI-2): week 48 results from two multicentre, double-blind, randomised, non-inferiority, phase 3 trials. Lancet. 2019;393(10167): 143–55. doi: 10.1016/S0140-6736(18)32462-0 30420123

[22] Aboud M, Orkin C, Podzamczer D, Bogner JR, Baker D, Khuong-Josses MA, et al. Efficacy and safety of dolutegravir-rilpivirine for maintenance of virological suppression in adults with HIV-1: 100-week data from the randomised, open-label, phase 3 SWORD-1 and SWORD-2 studies. Lancet HIV. 2019;6(9): e576–e87. doi: 10.1016/S2352-3018(19)30149-3 31307948

[23] Mayer KH, Loo S, Crawford PM, Crane HM, Leo M, DenOuden P, et al. Excess Clinical Comorbidity Among HIV-Infected Patients Accessing Primary Care in US Community Health Centers. Public Health Rep. 2018;133(1): 109–18. doi: 10.1177/0033354917748670 29262289 PMC5805107

[24] Iwamoto A, Taira R, Yokomaku Y, Koibuchi T, Rahman M, Izumi Y, et al. The HIV care cascade: Japanese perspectives. PLoS One. 2017;12(3): e0174360. doi: 10.1371/journal.pone.0174360 28319197 PMC5358866

[25] Miyazaki N, Sugiura W, Gatanaga H, Watanabe D, Yamamoto Y, Yokomaku Y, et al. The Prevalence of High Antiretroviral Coverage and Viral Suppression in Japan: an Excellent Profile for a Downstream Human Immunodeficiency Virus Care Spectrum. Jpn J Infect Dis. 2017;70(2): 158–60. doi: 10.7883/yoken.JJID.2015.599 27357985

[26] Ruzicka DJ, Imai K, Takahashi K, Naito T. Comorbidities and the use of comedications in people living with HIV on antiretroviral therapy in Japan: a cross-sectional study using a hospital claims database. BMJ Open. 2018;8(6): 2017–019985. doi: 10.1136/bmjopen-2017-019985 29903786 PMC6009456

[27] Badowski M, Pérez SE, Silva D, Lee A. Two’s a Company, Three’s a Crowd: A Review of Initiating or Switching to a Two-Drug Antiretroviral Regimen in Treatment-Naïve and Treatment-Experienced Patients Living with HIV-1. Infect Dis Ther. 2020;9(2): 185–208. doi: 10.1007/s40121-020-00290-w 32193799 PMC7237600

[28] Quan H, Li B, Couris CM, Fushimi K, Graham P, Hider P, et al. Updating and validating the Charlson comorbidity index and score for risk adjustment in hospital discharge abstracts using data from 6 countries. Am J Epidemiol. 2011;173(6): 676–82. doi: 10.1093/aje/kwq433 21330339

[29] AIDSinfo Glossary. [cited 2020 June 29]. Available from: https://aidsinfo.nih.gov/understanding-hiv-aids/glossary/1612/nrti-sparing-regimen.

[30] Pierone G, Henegar C, Fusco J, Vannappagari V, Aboud M, Ragone L, et al. Two-drug antiretroviral regimens: an assessment of virologic response and durability among treatment-experienced persons living with HIV in the OPERA(®) Observational Database. Journal of the International AIDS Society. 2019;22(12): e25418–e. doi: 10.1002/jia2.25418 31802641 PMC6893210

[31] Greenberg L, Ryom L, Neesgaard B, Wandeler G, Staub T, Gisinger M, et al. Clinical Outcomes of 2-Drug Regimens vs 3-Drug Regimens in Antiretroviral Treatment-Experienced People Living With Human Immunodeficiency Virus. Clin Infect Dis. 2021;73(7): e2323–e33. doi: 10.1093/cid/ciaa1878 33354721 PMC9431658

[32] Neesgaard B, Pelchen-Matthews A, Ryom L, Florence E, Peters L, Roen A, et al. Uptake and effectiveness of two-drug compared with three-drug antiretroviral regimens among HIV-positive individuals in Europe. Aids. 2019;33(13): 2013–24. doi: 10.1097/QAD.0000000000002320 31335807

[33] de Lazzari E, Gonzalez-Cordon A, Inciarte A, Ugarte A, de la Mora L, Martinez-Rebollar M, et al. Factors associated with the use and composition of two-drug regimens in a large single-centre HIV cohort. J Antimicrob Chemother. 2021;76(11): 2988–92. doi: 10.1093/jac/dkab261 34293162

[34] Serrano-Villar S, Gutierrez F, Miralles C, Berenguer J, Rivero A, Martinez E, et al. Human Immunodeficiency Virus as a Chronic Disease: Evaluation and Management of Nonacquired Immune Deficiency Syndrome-Defining Conditions. Open Forum Infect Dis. 2016;3(2). doi: 10.1093/ofid/ofw097 27419169 PMC4943534

[35] Nishijima T, Komatsu H, Gatanaga H, Aoki T, Watanabe K, Kinai E, et al. Impact of small body weight on tenofovir-associated renal dysfunction in HIV-infected patients: a retrospective cohort study of Japanese patients. PLoS One. 2011;6(7): e22661. doi: 10.1371/journal.pone.0022661 21799928 PMC3143186

[36] Mizushima D, Tanuma J, Kanaya F, Nishijima T, Gatanaga H, Lam NT, et al. WHO antiretroviral therapy guidelines 2010 and impact of tenofovir on chronic kidney disease in Vietnamese HIV-infected patients. PLoS One. 2013;8(11): e79885. doi: 10.1371/journal.pone.0079885 24223203 PMC3819298

[37] Nishijima T, Komatsu H, Higasa K, Takano M, Tsuchiya K, Hayashida T, et al. Single nucleotide polymorphisms in ABCC2 associate with tenofovir-induced kidney tubular dysfunction in Japanese patients with HIV-1 infection: a pharmacogenetic study. Clin Infect Dis. 2012;55(11): 1558–67. doi: 10.1093/cid/cis772 22955427

[38] Nishijima T, Mutoh Y, Kawasaki Y, Tomonari K, Kikuchi Y, Gatanaga H, et al. Cumulative exposure of TDF is associated with kidney tubulopathy whether it is currently used or discontinued. AIDS. 2018;32(2): 179–88. doi: 10.1097/QAD.0000000000001667 29028660

[39] Raffi F, Babiker AG, Richert L, Molina JM, George EC, Antinori A, et al. Ritonavir-boosted darunavir combined with raltegravir or tenofovir-emtricitabine in antiretroviral-naive adults infected with HIV-1: 96 week results from the NEAT001/ANRS143 randomised non-inferiority trial. Lancet. 2014;384(9958): 1942–51. doi: 10.1016/S0140-6736(14)61170-3 25103176

[40] Baril JG, Angel JB, Gill MJ, Gathe J, Cahn P, van Wyk J, et al. Dual Therapy Treatment Strategies for the Management of Patients Infected with HIV: A Systematic Review of Current Evidence in ARV-Naive or ARV-Experienced, Virologically Suppressed Patients. PLoS One. 2016;11(2): e0148231. doi: 10.1371/journal.pone.0148231 26849060 PMC4746196

[41] Moreno S, Perno CF, Mallon PW, Behrens G, Corbeau P, Routy JP, et al. Two-drug vs. three-drug combinations for HIV-1: Do we have enough data to make the switch? HIV Med. 2019;20 Suppl 4: 2–12. doi: 10.1111/hiv.12716 30821898

[42] Taiwo B, Zheng L, Gallien S, Matining RM, Kuritzkes DR, Wilson CC, et al. Efficacy of a nucleoside-sparing regimen of darunavir/ritonavir plus raltegravir in treatment-naive HIV-1-infected patients (ACTG A5262). AIDS. 2011;25(17): 2113–22. doi: 10.1097/QAD.0b013e32834bbaa9 21857490 PMC3515052

