# Supplementary material for: Characteristics of 2-drug regimen users living with HIV-1 in a real-world setting: A large-scale medical claim database analysis in Japan
Source: PLoS One. 2022 Jun 14;17(6):e0269779. doi: 10.1371/journal.pone.0269779 (PMC9197042; doi:10.1371/journal.pone.0269779)
Supplement: S1 Table — (DOCX) [file pone.0269779.s001.docx]

**S1 Table.** Rank of co-medications

| **Rank** | **2-Drug** | | | | **3-Drug** | | |
| --- | --- | --- | --- | --- | --- | --- | --- |
|  |  | **ATC4 name** | **N** | **%** | **ATC name** | **N** | **%** |
| **1** |  | Proton pump inhibitors | 36 | 38.30% | Systemic antihistamines | 1130 | 28.30% |
| **2** |  | Angiotensin-II antagonists, plain | 27 | 28.72% | Non-barbiturates, plain | 765 | 19.16% |
| **3** |  | Calcium antagonists, plain | 25 | 26.60% | Proton pump inhibitors | 665 | 16.65% |
| **4** |  | Anti-gout preparations | 24 | 25.53% | Anti-rheumatic, non-steroidal plain | 652 | 16.33% |
| **5** |  | Non-barbiturates, plain | 23 | 24.47% | All other anti-ulcerants | 489 | 12.25% |
| **6** |  | Statins (HMG-CoA reductase inhibitors) | 23 | 24.47% | Non-narcotics and anti-pyretics | 419 | 10.49% |
| **7** |  | Non-narcotics and anti-pyretics | 22 | 23.40% | Anti-gout preparations | 414 | 10.37% |
| **8** |  | Systemic antihistamines | 22 | 23.40% | Statins (HMG-CoA reductase inhibitors) | 412 | 10.32% |
| **9** |  | Trimethoprim and similar formulations | 17 | 18.09% | Trimethoprim and similar formulations | 404 | 10.12% |
| **10** |  | Loop diuretics plain | 17 | 18.09% | Anti-diarrhoeal micro-organisms | 400 | 10.02% |
| **11** |  | Plain antacids | 16 | 17.02% | Angiotensin-II antagonists, plain | 398 | 9.97% |
| **12** |  | Anti-rheumatic, non-steroidal plain | 16 | 17.02% | Tranquillizers | 382 | 9.57% |
| **13** |  | Anti-diarrhoeal micro-organisms | 15 | 15.96% | Calcium antagonists, plain | 273 | 6.84% |
| **14** |  | All other anti-ulcerants | 15 | 15.96% | Kanpo medicines | 270 | 6.76% |
| **15** |  | H2 antagonists | 13 | 13.83% | H2 antagonists | 267 | 6.69% |
