# Supplementary material for: Characteristics of 2-drug regimen users living with HIV-1 in a real-world setting: A large-scale medical claim database analysis in Japan
Source: PLoS One. 2022 Jun 14;17(6):e0269779. doi: 10.1371/journal.pone.0269779 (PMC9197042; doi:10.1371/journal.pone.0269779)
Supplement: S2 Table — Age was included as a continuous variable and the corresponding OR represents per 1-year increase. (DOCX) [file pone.0269779.s002.docx]

**S2 Table.** Adjusted analysis of co-medications and comorbidities

| *Outcome: any co-medications* |  | Odds Ratio | |  |
| --- | --- | --- | --- | --- |
|  | Odds Ratio | Lower limit | Upper limit | P-value |
| **2-drug cohort vs 3-drug cohort** | 2.59 | 1.18 | 5.68 | **0.0179** |
| Age | 1.04 | 1.04 | 1.05 | <0.0001 |
| Gender: Male vs Female | 1.01 | 0.73 | 1.40 | 0.9465 |
| Year of index date (n, %) |  |  |  |  |
| Cluster 1 (2008 Q2- 2014 Q3) | *Ref* |  |  |  |
| Cluster 2 (2014 Q4- 2016 Q2) | 0.73 | 0.54 | 0.98 | 0.0365 |
| Cluster 3 (2016 Q3 - last available data) | 0.41 | 0.34 | 0.50 | <0.0001 |
| *Outcome: any AIDS-defining conditions* |  | Odds Ratio | |  |
|  | Odds Ratio | Lower limit | Upper limit | P-value |
| **2-drug cohort vs 3-drug cohort** | 1.73 | 1.09 | 2.74 | **0.0203** |
| Age | 1.03 | 1.03 | 1.04 | <0.0001 |
| Gender: Male vs Female | 0.82 | 0.62 | 1.07 | 0.1421 |
| Year of index date (n, %) |  |  |  |  |
| Cluster 1 (2008 Q2- 2014 Q3) | *Ref* |  |  |  |
| Cluster 2 (2014 Q4- 2016 Q2) | 0.92 | 0.73 | 1.16 | 0.4757 |
| Cluster 3 (2016 Q3 - last available data) | 0.26 | 0.22 | 0.30 | <0.0001 |
| *Outcome: Charlson Comorbidity Score* | Parameter Estimate | Lower limit | Upper limit | P-value |
| **2-drug cohort vs 3-drug cohort** | 1.17 | 0.84 | 1.50 | <**0.0001** |
| Age | 0.03 | 0.03 | 0.04 | <0.0001 |
| Gender: Male vs Female | 0.26 | 0.06 | 0.46 | 0.0115 |
| Year of index date (n, %) |  |  |  |  |
| Cluster 1 (2008 Q2- 2014 Q3) | *Ref* |  |  |  |
| Cluster 2 (2014 Q4- 2016 Q2) | -0.07 | -0.25 | 0.10 | 0.4203 |
| Cluster 3 (2016 Q3 - last available data) | -0.29 | -0.41 | -0.18 | <0.0001 |
