# Supplementary material for: Characteristics of 2-drug regimen users living with HIV-1 in a real-world setting: A large-scale medical claim database analysis in Japan
Source: PLoS One. 2022 Jun 14;17(6):e0269779. doi: 10.1371/journal.pone.0269779 (PMC9197042; doi:10.1371/journal.pone.0269779)
Supplement: S3 Table — All conditions were identified using ICD-10 codes or Japanese disease codes. (DOCX) [file pone.0269779.s003.docx]

**S3 Table.** AIDS-defining conditions among each cohort

|  | **2-Drug** | | **3-Drug** | |
| --- | --- | --- | --- | --- |
|  | N | % | N | % |
| Individuals with any AIDS-defining condition | 62 | 66.0% | 1708 | 42.8% |
| Individuals with any AIDS-defining condition, except AIDS | 40 | 42.6% | 1262 | 31.6% |
| AIDS-defining conditions |  |  |  |  |
| AIDS | 49 | 52.1% | 991 | 24.8% |
| Cytomegalovirus disease | 16 | 17.0% | 418 | 10.5% |
| Encephalopathy | 4 | 4.3% | 99 | 2.5% |
| Wasting syndrome | 0 | 0.0% | 0 | 0.0% |
| Coccidiodomycosis | 0 | 0.0% | 0 | 0.0% |
| Cryptococcosis | 3 | 3.2% | 72 | 1.8% |
| Cryptosporidiosis | 0 | 0.0% | 1 | 0.0% |
| Histoplasmosis | 0 | 0.0% | 0 | 0.0% |
| Isosporiasis | 0 | 0.0% | 0 | 0.0% |
| Pulmonary mycobacterial infection | 2 | 2.1% | 37 | 0.9% |
| Tuberculosis | 6 | 6.4% | 255 | 6.4% |
| Mycobacterial Infection | 0 | 0.0% | 4 | 0.1% |
| Pneumocsystosis | 19 | 20.2% | 881 | 22.1% |
| Progressive multifocal leukoencephalopathy | 1 | 1.1% | 30 | 0.8% |
| Salmonella septicaemia | 0 | 0.0% | 1 | 0.0% |
| Toxoplasma meningoencephalitis | 2 | 2.1% | 22 | 0.6% |
| Burkitt lymphoma | 0 | 0.0% | 9 | 0.2% |
| Kaposi sarcoma | 2 | 2.1% | 69 | 1.7% |
| Non-Hodgkin lymphoma | 4 | 4.3% | 127 | 3.2% |
| Cancer of the cervix uteri | 0 | 0.0% | 4 | 0.1% |
