# Supplementary material for: Characteristics of 2-drug regimen users living with HIV-1 in a real-world setting: A large-scale medical claim database analysis in Japan
Source: PLoS One. 2022 Jun 14;17(6):e0269779. doi: 10.1371/journal.pone.0269779 (PMC9197042; doi:10.1371/journal.pone.0269779)
Supplement: S4 Table — All regimens listed above may or may not be used with an additional booster; *Tenofovir includes tenofovir disoproxil fumarate and tenofovir alafenamide fumarate. (DOCX) [file pone.0269779.s004.docx]

**S4 Table.** Top 10 regimen in 2- and 3-drug regimen cohort

| **2-Drug (N=94)** | | | |
| --- | --- | --- | --- |
| **Rank** | **Regimen** | **N** | **%** |
| 1 | Darunavir ethanolate, raltegravir potassium | 20 | 21.28% |
| 2 | Dolutegravir sodium, rilpivirine hydrochloride | 13 | 13.83% |
| 3 | Darunavir ethanolate, dolutegravir sodium | 7 | 7.45% |
| 4 | Etravirine, raltegravir potassium | 7 | 7.45% |
| 5 | Efavirenz, raltegravir potassium | 6 | 6.38% |
| 6 | Darunavir ethanolate, rilpivirine hydrochloride | 4 | 4.26% |
| 7 | Abacavir sulfate, efavirenz | 3 | 3.19% |
| 8 | Abacavir sulfate, raltegravir potassium | 3 | 3.19% |
| 9 | Dolutegravir sodium, lamivudine | 3 | 3.19% |
| 10 | Lopinavir, raltegravir potassium | 3 | 3.19% |
|  | | | |
| **3-Drug (N=3,993)** | | | |
| **Rank** | **Regimen** | **N** | **%** |
| 1 | Dolutegravir sodium, emtricitabine, tenofovir* | 820 | 20.54% |
| 2 | Abacavir sulfate, dolutegravir sodium, lamivudine | 764 | 19.13% |
| 3 | Emtricitabine, raltegravir potassium, tenofovir* | 461 | 11.55% |
| 4 | Elvitegravir, emtricitabine, tenofovir* | 371 | 9.29% |
| 5 | Darunavir ethanolate, emtricitabine, tenofovir* | 347 | 8.69% |
| 6 | Efavirenz, emtricitabine, tenofovir* | 267 | 6.69% |
| 7 | Abacavir sulfate, lamivudine, raltegravir potassium | 231 | 5.79% |
| 8 | Abacavir sulfate, efavirenz, lamivudine | 115 | 2.88% |
| 9 | Abacavir sulfate, darunavir ethanolate, lamivudine | 97 | 2.43% |
| 10 | Atazanavir sulfate, emtricitabine, tenofovir* | 84 | 2.10% |

*All regimens listed above may or may not be used with an additional booster*

**Tenofovir includes tenofovir disoproxil fumarate and tenofovir alafenamide fumarate*
