# Supplementary figures and images for: Characteristics of 2-drug regimen users living with HIV-1 in a real-world setting: A large-scale medical claim database analysis in Japan
Source: PLoS One. 2022 Jun 14;17(6):e0269779. doi: 10.1371/journal.pone.0269779 (PMC9197042; doi:10.1371/journal.pone.0269779)

**
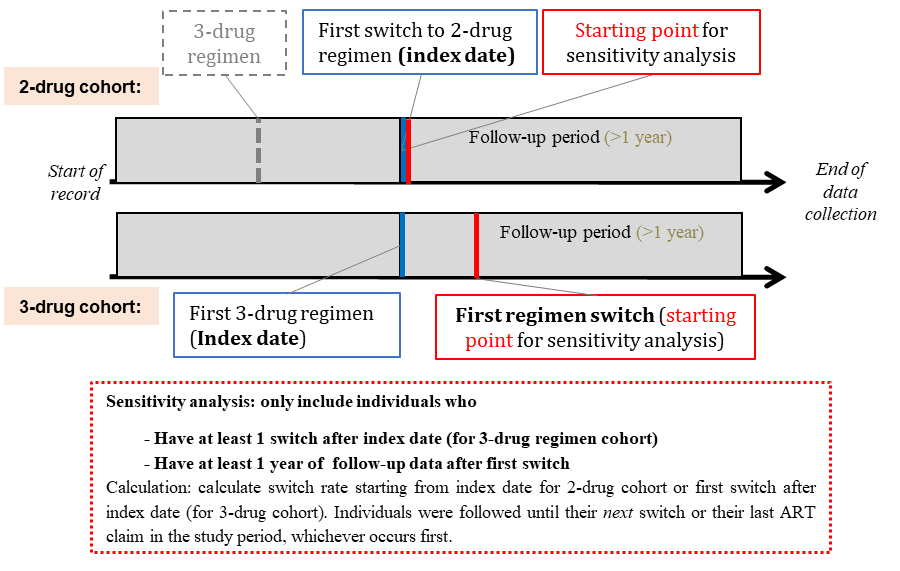
 S1 Fig.** Study Design

Supplement: S1 Fig — (DOCX) [file pone.0269779.s005.docx]

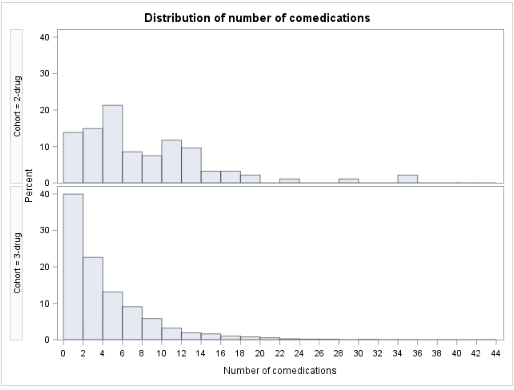


**S2 Fig.** Number of co-medications

Supplement: S2 Fig — (DOCX) [file pone.0269779.s006.docx]

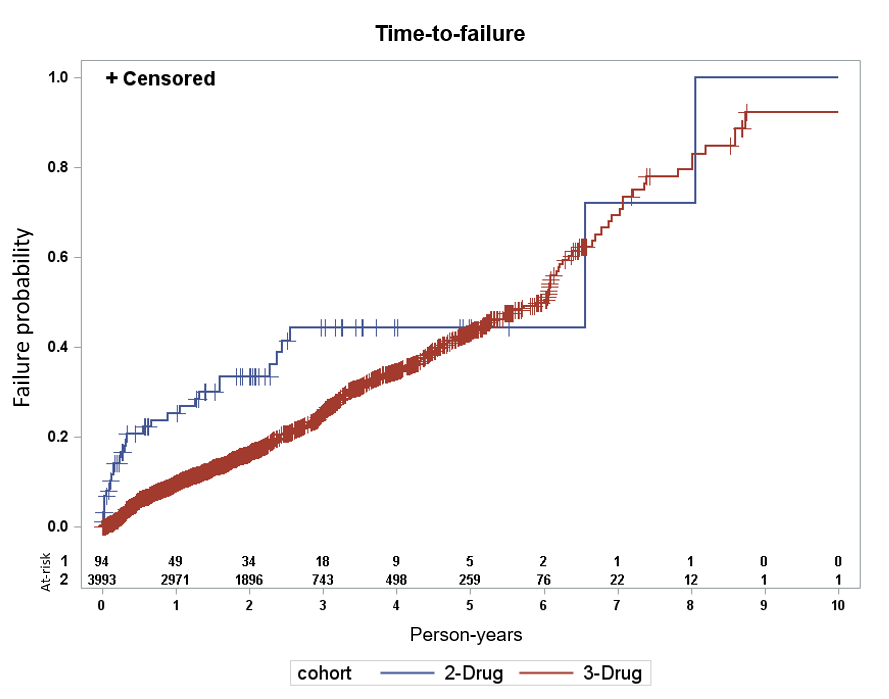


**S3 Fig.** Kaplan Meier curve for time-to-switch for the 2- and 3-drug regimen cohorts

Supplement: S3 Fig — (DOCX) [file pone.0269779.s007.docx]

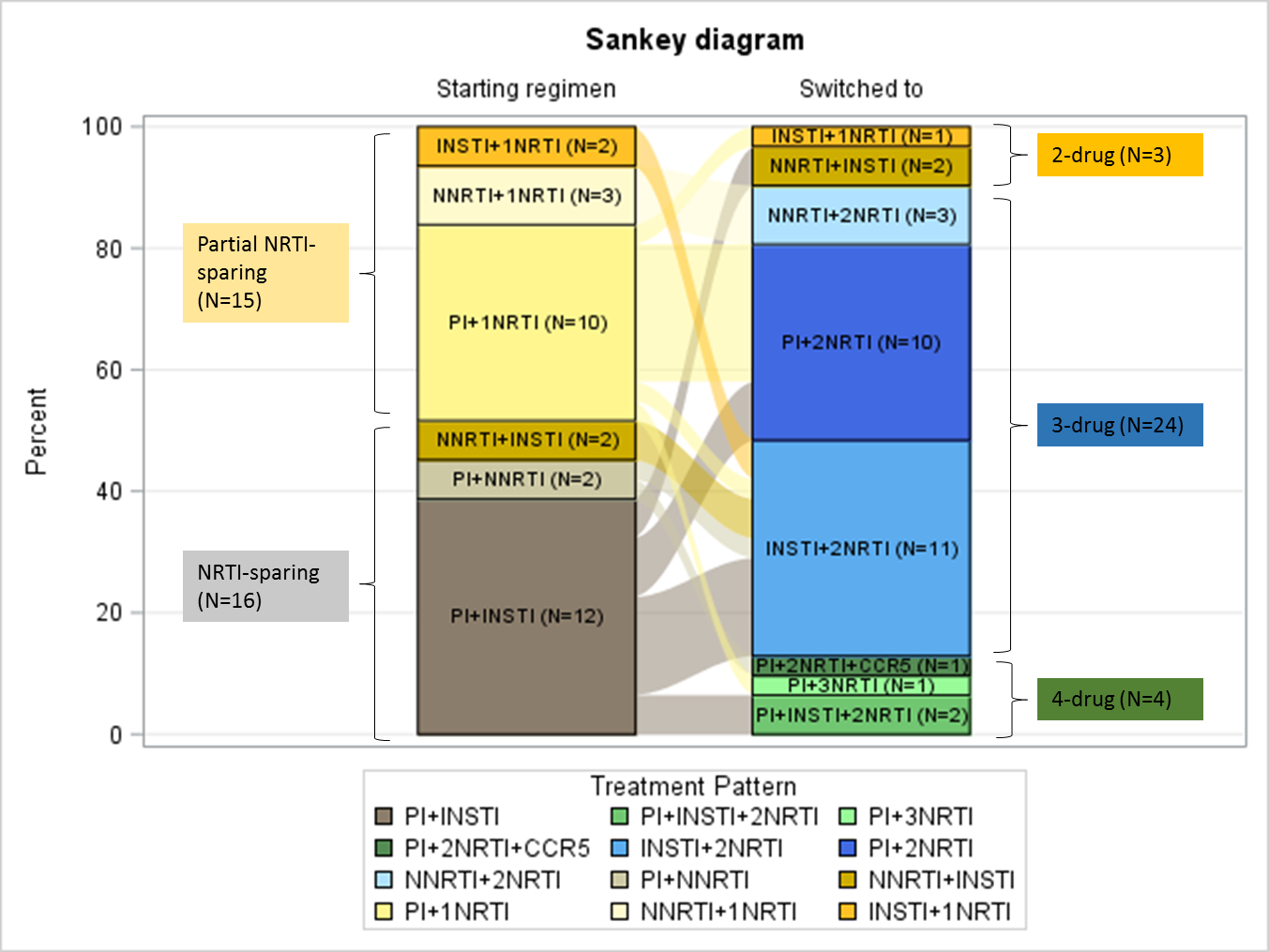


**S4 Fig.** Switch patterns for 2-drug regimen cohort

Supplement: S4 Fig — (DOCX) [file pone.0269779.s008.docx]
